# Lipid nanoparticles and extracellular vesicles: emerging cell free therapeutic platforms for hepatobiliary cancers

**DOI:** 10.3389/fcell.2026.1812373

**Published:** 2026-05-28

**Authors:** Gabriele Saccu, Sharmila Fagoonee

**Affiliations:** 1 Boston Children’s Hospital, Pathology Department, Harvard University, Boston, MA, United States; 2 Institute of Biostructure and Bioimaging (CNR) and Molecular Biotechnology Center “Guido Tarone”, Turin, Italy

**Keywords:** biomarkers, cancer therapy, drug delivery, extracellular vesicles, hepatobiliary cancers, lipid nanoparticles, mRNA vaccine

## Abstract

Hepatobiliary cancers are major contributors to cancer-related mortality, mainly due to late-stage diagnosis, poor prognosis, and limited effectiveness of current treatments in advanced disease. Standard therapies comprise surgical resection, liver transplantation, and systemic agents including tyrosine kinase inhibitors and immune checkpoint inhibitors, but their efficacy is often hindered by the development of drug resistance, underlying the urgent need for novel therapeutic strategies. Lipid nanoparticles (LNPs) have emerged as promising delivery vehicles due to their favorable physicochemical properties, enabling the transport of natural biomolecules or engineered drugs for targeted tumor therapy. LNPs are also being explored as platforms for cancer therapy and vaccines. Moreover, recent advances underscore the key role of extracellular vesicles (EVs) in modulating the tumor microenvironment, promoting tumor progression, chemoresistance, and escape from immune recognition and destruction (immune evasion) in hepatobiliary cancers. EVs are being investigated both as biomarkers for early detection and as therapeutic agents, with engineered or drug-loaded EVs showing potential for targeted drug delivery and tissue repair in liver diseases, including cancer. This review highlights the potential of cell-free therapeutic LNP- and EV-based platforms, standalone or in combination, for RNA, small molecules and proteins delivery in improving diagnosis and treatment outcomes in hepatobiliary cancers, in particular, hepatocellular carcinoma and cholangiocarcinoma.

## 1 Introduction

Human hepatobiliary cancers encompass aggressive malignancies that originate in the liver, bile ducts and gallbladder, comprising hepatocellular carcinoma (HCC), cholangiocarcinoma (CCA), gallbladder carcinoma (GBC). HCC originates in the liver cells while CCA, which arises from the bile ducts, is further classified as intrahepatic or extrahepatic (Spencer et al., 2023). Collectively, CCAs and gallbladder cancer are often referred to as biliary tract cancers (BTCs). The lack of specific symptoms in early stages of hepatobiliary tumors leads to diagnosis mostly at advanced stages, contributing to high morbidity and mortality worldwide (Kim et al., 2024). The main causes include hepatitis B and C, alcohol use, chronic biliary tract inflammation, genetic and metabolic diseases, with the burden varying significantly by region and socioeconomic status (Khan et al., 2019; Llovet et al., 2021). Hepatobiliary cancers are characterized by significant molecular heterogeneity resulting in activation of distinct patterns of biological pathway activation (Roy et al., 2025). Comparative transcriptomic analyses show that HCC is particularly associated with immune system modulation, while CCA is characterized by metabolic dysregulation, especially in lipid metabolism, and GBC is linked to cell cycle regulation (Roy et al., 2025; Yang F. et al., 2023).

As cancers of the hepatobiliary system are relatively uncommon, progress in developing effective therapeutic strategies has been slow. The rarity of these malignancies has hampered both clinical experience and research opportunities, making it challenging to establish standardized treatment protocols (Pant and Gradilone, 2022). The high metastatic potential severely limits the number of patients who are suitable candidates for potentially curative interventions, further complicating this issue (Gunasekaran et al., 2021). Due to late diagnosis, surgical treatment, the mainstay for curative intent, is frequently complex, highly invasive, and associated with considerable perioperative risks, especially given the underlying liver dysfunction common in these patients (Gunasekaran et al., 2021; Lauterio et al., 2021; Chakraborty and Sarkar, 2022). Additionally, intrinsic and acquired drug resistance, along with dense stromal microenvironments, especially in CCA, limit the efficacy of conventional chemotherapy and contribute to systemic toxicity. As a consequence, outcomes for patients with hepatobiliary cancers remain generally poor, reflecting the combined impact of tumor aggressiveness, diagnostic challenges, and treatment-related limitations (Tsilimigras et al., 2025). These factors emphasize the urgent need for innovative approaches and more effective therapies to improve prognosis in this patient population.

In recent years, the therapeutic landscape for hepatobiliary cancers has undergone remarkable advancements. The emergence of molecularly targeted agents and immunotherapies for HCC has clearly reshaped the management of advanced-stage disease, offering new options for patients who had limited prospects earlier (Zhang et al., 2022; Philippi et al., 2025). At the same time, the optimization and widespread adoption of minimally invasive laparoscopic surgical techniques have expanded the cohort of patients eligible for operative intervention, potentially allowing more individuals to benefit from surgical resection while reducing procedure-related morbidity (Kato et al., 2023). The criteria for liver transplantation in the framework of hepatobiliary malignancies have also gradually broadened, increasingly placing transplantation as a viable, and in some cases, the only, therapeutic option for patients with otherwise terminal disease (Gorji et al., 2024). Collectively, these innovations highlight an important shift toward more personalized, less invasive, and potentially life-extending treatment strategies for patients facing hepatobiliary cancers (Tsilimigras et al., 2025; Zhang et al., 2022; Yang C. et al., 2023).

Novel therapies for hepatobiliary cancers increasingly focus on RNA (mRNA or circular RNA) vaccine technology, which presents a promising immunotherapeutic approach by inducing the immune system to recognize and attack tumor-specific antigens. In contrast to conventional small-molecule drugs or monoclonal antibodies that target proteins, RNA-based therapies functions upstream at the level of gene expression, offering the potential to modulate previously “undruggable” targets. RNA-based therapies portray a promising approach for hepatobiliary cancers, as they allow direct control of gene expression within tumor cells, either by stimulating the production of therapeutic proteins (as in mRNA-based approaches) or by silencing disease-driving genes (as in RNA interference strategies such as small interference RNAs or siRNAs and microRNAs or miRNAs) (Yuan et al., 2025; Xing et al., 2024). Nevertheless, a major challenge in cancer therapy, especially for nucleic acid-based drugs, is the presence of multiple biological barriers, including enzymatic degradation in the bloodstream, limited entry into target cells (cellular uptake), and inefficient intracellular release, all of which significantly reduce therapeutic efficacy (Zhang L. et al., 2024). Cell-free therapeutic strategies are garnering growing interest in oncology as they enable the delivery of functional biomolecules without requiring living cells, thereby circumventing many of the challenges associated with cell-based therapies, such as complex manufacturing, safety concerns, and limited scalability (de Almeida Fuzeta et al., 2020). In this context, lipid-based nanocarriers, such as lipid nanoparticles (LNPs) and extracellular vesicles (EVs) represent two complementary platforms for RNA delivery, providing protection for the mRNA, facilitating cellular uptake, enhancing endosomal escape, and enabling immune activation. Molecules such as mRNA and siRNA must reach the cytosol to be translated or to engage the RNA interference machinery in order to exert therapeutic activity. Once engulfed by cells, the nanocarrier containing these biomolecules becomes entrapped inside endosomes, and can undergo degradation in lysosomes. For the therapy to work, the therapeutic mRNA must escape from these vesicles into the cytoplasm before degradation occurs, a process termed endosomal escape.

LNPs are synthetic lipid-based carriers engineered for drug delivery, whereas EVs are naturally released cell-derived particles that mediate intercellular communication. Regarding LNPs, the world has recently witnessed how mRNA vaccines, such as Moderna’s COVID-19 vaccine, utilizing LNPs to deliver mRNA encoding antigens, have revolutionized infectious disease prevention. However, their adaptation for cancer immunotherapy is a promising but still developing field, with challenges including delivery optimization and immune modulation remaining to be addressed (Miao et al., 2021; Kon et al., 2023; Ramadan et al., 2024). Additionally, the exploration of alternative nanocarriers, including polymers, peptides, and EVs, aims to further improve biocompatibility and targeting (Miao et al., 2021).

In spite of the rapid expansion of research on nanocarrier-based RNA delivery, LNPs and EVs are currently evaluated as distinct and independent platforms, with limited integration of their complementary biological and technological features. Moreover, few reviews have specifically addressed their application within the unique biological and clinical context of hepatobiliary malignancies, which are characterized by a highly immunosuppressive tumor microenvironment (TME) and significant inter-tumoral heterogeneity. In this review, we present an integrative perspective on LNP- and EV-based delivery systems for therapeutic biomolecules in hepatobiliary cancers. We highlight their distinct yet complementary features, in terms of biogenesis, membrane dynamics and interactions with the tumor microenvironment. Furthermore, we examine LNPs and EVs within a unified cell-free framework as emerging hybrid approaches with emerging clinical relevance for hepatobiliary cancers. The TME, composed of stromal cells, immune cells, extracellular matrix, and soluble factors, plays a critical role in tumor progression and therapy resistance by generating a physical and immunosuppressive barrier to effective treatment. We further underscore the interplay between nanocarrier design, TME modulation, and immune activation, linking mechanistic insights to translational and clinical developments. By consolidating these aspects, the present review aims to provide a coherent framework to guide the rational design of next-generation therapeutics, including RNA, proteins and small molecules, for hepatobiliary malignancies.

## 2 Current treatment strategies for hepatobiliary cancers

Treatment strategies for hepatobiliary cancers depend on a multidisciplinary approach involving surgery, transplantation, locoregional therapies, systemic treatments, and emerging molecular-targeted and immunotherapies (Table 1). Surgical resection remains the gold standard for early-stage disease, with liver transplantation and ablation as alternatives for selected HCC patients (Vitale et al., 2024). On the other hand, adjuvant chemotherapy and radiotherapy have uncertain but potentially beneficial roles in advanced cases (Gunasekaran et al., 2021; Benson et al., 2021). To this regard, systemic therapy has evolved from sorafenib (a tyrosine kinase inhibitor) monotherapy to immunotherapy combinations such as atezolizumab plus bevacizumab, which have demonstrated superior survival benefits and are now frontline standards (Chakraborty and Sarkar, 2022). Atezolizumab is a monoclonal antibody targeting programmed death-ligand 1 (PD-L1), while bevacizumab inhibits vascular endothelial growth factor (VEGF), a key driver of tumor angiogenesis. By combining immune activation (through PD-L1 blockade) and tumor blood vessel formation, this regimen promotes T cell–mediated anti-tumor responses and improves drug delivery within the TME, resulting in superior survival outcomes with respect to previous standard therapies. BTCs have witnessed progress with capecitabine as adjuvant therapy, which stimulate the innate immune system and enhance antigen-specific immune responses, and new chemotherapy combinations improving survival in advanced stages, alongside targeted therapies guided by molecular profiling, which is increasingly important given the heterogeneity and actionable mutations in these cancers (Tsilimigras et al., 2025; Oneda et al., 2020; Guven et al., 2023). Of interest, locoregional treatments like trans-arterial chemoembolization, radioembolization, and hepatic arterial infusion chemotherapy (HAIC) offer valuable options especially for unresectable or advanced tumors, with HAIC showing promise in recent trials for both HCC and CCA (Jipa and Makary, 2024; Zheng and Wang, 2024). These approaches rely on achieving high local drug concentrations while minimizing systemic toxicity, representing a viable alternative for hepatobiliary cancer patients who are not eligible for surgery.

**TABLE 1:** Examples of current clinical treatment strategies for hepatobiliary cancers highlighting modes of administration, patient populations and cancer types, and clinical trial references, if available.

Treatment strategy	Mode of administration	Patient and cancer types	Clinical trials	Reference(s)
Molecular targeted therapies (such as HER2, NTRK, RET, BRAF inhibitors)	Oral or intravenous, depending on drug	Advanced BTCs and some HCC	not specified	Tsilimigras et al. (2025), Tsumura et al. (2022)
Immunotherapy (immune checkpoint inhibitors alone or combined with chemotherapy)	Intravenous infusion	Advanced HCC and BTC patients	KEYNOTE-966, TOPAZ-1, GEMINI-Hepatobiliary	Guven et al. (2023), Wheless et al. (2024), Zhou et al. (2024b)
Traditional Chinese Medicine (e.g., Huaier Granule, Huachansu, Icaritin)	Oral or injection	Hepatic, biliary, and pancreatic cancer patients	not specified	Wu et al. (2024)
Cellular immunotherapy (e.g., CAR-T, receptor-engineered T cells)	Intravenous infusion	Patients with hepatobiliary malignancies (HCC and BTC)	not specified	Xue et al. (2022)
Adjuvant chemotherapy (e.g., capecitabine)	Oral	Post-surgical BTC patients	Standard of care, clinical trials ongoing	Guven et al. (2023), Wheless et al. (2024)
Combination gemcitabine and cisplatin with immune checkpoint blockade	Intravenous infusion	Advanced BTC patients	KEYNOTE-966, TOPAZ-1	Wheless et al. (2024), Zhou et al. (2024b)

In this scenario, immunotherapy approaches are reshaping treatment paradigms for both HCC and BTCs, leading to improved outcomes and becoming integral to first-line and subsequent therapies, despite the fact that optimal clinical treatment sequencing strategies and biomarker-driven patient selection remain areas of active research (Cabibbo et al., 2025; Vogel et al., 2022). Among immunotherapy strategies, immune checkpoint inhibitors (ICIs) have shown promising results by enhancing the immune system’s ability to eradicate cancer cells. They act by releasing “brakes” on the immune system and restoring cytotoxic T cell function. The clinical benefit of ICIs, administered as monotherapy or in combination regimens (e.g., anti–PD-L1 plus anti-VEGF), represents a feasible treatment strategy by enhancing T cell–mediated anti-tumor responses, ultimately leading to durable clinical responses in patients (Mc Neil and Lee, 2025).

Building on these approaches, RNA-based strategies, including mRNA vaccines, represent a novel immunotherapeutic strategy that can specifically activate the immune system against tumor antigens by delivering mRNA via LNPs, offering high potency and versatility that may complement existing treatments and improve outcomes in hepatobiliary cancers (discussed below). Likewise, preclinical studies have investigated the role of RNA cargos by EVs in HCC and CCA, highlighting EVs as a promising therapeutic platform (Huang Z. et al., 2025; Li et al., 2017). Their engineering potential may enable highly specific liver tumor targeting and may further enhance therapeutic efficacy.

Some biological differences between HCC and CCA, summarized in Table 2, exist and need to be taken in consideration in designing the best nanocarrier-based strategies. While HCC nanocarrier strategies aim mainly at molecular targeting and resistance bypass (that is, identify and penetrate the right cells), CCA nanocarrier strategies emphasize stroma penetration and TME reprogramming (in other words, strategies must first remodel the structurally and biologically restrictive environment to pass through and reach the tumor cells). In CCA, the TME is very dense, fibrotic stroma composed of cancer-associated fibroblasts, extracellular matrix proteins, immune cells, and abnormal vasculature, acting as both a physical barrier and a biological barrier; on the other hand, in HCC, the TME is more cellular and better vascularized (Banales et al., 2020; Argentiero et al., 2023).

**TABLE 2:** Biological differences between HCC and CCA.

Feature	HCC	CCA
Cell of origin	Hepatocytes	Cholangiocytes
Architecture	Highly vascular	Dense, fibrotic (desmoplastic)
Immune milieu	Immunotolerant, inflamed	Profoundly immunosuppressive
Drug delivery barriers	Efflux pumps, heterogeneity	Physical stromal barrier
Clinical therapy	TKIs, ICIs	Chemotherapy, limited targeted options

## 3 RNA therapeutics

RNA therapies make use of several types of RNA molecules, each with distinct mechanisms and therapeutic goals. RNA-based therapeutics for cancers are advancing but remain mainly preclinical or early clinical stages for hepatobiliary cancers like CCA (Table 3) (Fagoonee and Weiskirchen, 2024). On the other hand, RNA-based therapies for HCC are actively being explored both at preclinical levels and in clinical trials, with encouraging treatment outcomes. For instance, microRNAs (miRNAs), which are 20–25 nucleotides long RNA molecules acting as fine-tuners of gene activity, are widely studied for their ability to regulate oncogenes and tumor suppressors, with delivery systems including viral vectors, LNPs, and EVs designed to improve stability and targeting to tumor cells, such as those of HCC (Roy et al., 2022; Ishiguro et al., 2020). Small interfering RNAs (siRNAs), which are short, double-stranded, 20–25 nucleotides long RNA molecules and act like precise “off switches” for genes, are used to silence specific oncogenic transcripts, such as β-catenin in liver cancer stem cells, often delivered via engineered biological nanoparticles for targeted therapy (Xing et al., 2024). mRNA therapies include cancer vaccines that expose the immune system to tumor-associated antigens, helping it recognize cancer cells as abnormal and “train” the immune system to better detect and eliminate cancer cells, as well as protein restoration strategies, which are capable of fixing or replacing defective proteins, assisting cells reacquire normal function, with advances in nanocarrier delivery systems like LNPs enhancing their precision and efficacy (Yuan et al., 2025). Emerging non-coding RNAs such as piwi-interacting RNAs (piRNAs) have also shown tumor-suppressive effects in preclinical HCC models, representing a novel therapeutic avenue (Han et al., 2025). Additionally, long non-coding RNAs (lncRNAs) are targeted or used as therapeutic agents themselves, with nanoplatforms delivering siRNAs against oncogenic lncRNAs such as LINC00958 to inhibit tumor progression (Zuo et al., 2020; He et al., 2025). Overall, RNA nanotherapeutics leverage diverse RNA types, miRNA, siRNA, mRNA, piRNA, and lncRNA, to modulate gene expression, activate immune responses, and overcome drug resistance in hepatobiliary cancers (Vianello et al., 2024; Tian et al., 2024).

**TABLE 3:** Examples of RNA therapies for hepatobiliary cancers.

RNA therapy	Mode of administration	Patient types	Hepatobiliary cancer type	Clinical trial number	Reference(s)
MTL-CEBPA (small activating RNA)	Intravenous infusion, weekly cycles	Advanced HCC with cirrhosis, NASH, or liver metastases	HCC	NCT number not specified (Phase I trial)	Sarker et al. (2020)
RNA-based cancer vaccines and immunotherapies	Various (including injection)	Advanced HCC patients, often combined with immune checkpoint inhibitors	HCC	Various ongoing trials (not specified)	Xing et al. (2024), Vianello et al. (2024), Afra et al. (2023)
Lipid nanoparticle-delivered piRNA (piR-37213)	Systemic delivery *via* tail vein injection (twice weekly)	Preclinical animal models; human trials not yet started	HCC	Preclinical stage	Han et al. (2025)
PLGA-based nanoplatform delivering siRNA against LINC00958	Systemic administration (nanoparticle delivery)	Preclinical models; patient-derived xenografts	HCC	Preclinical stage	Zuo et al. (2020)
MiRNA and siRNA therapies	Various including LNPs and viral vectors	Advanced HCC patients in early clinical trials	HCC	Early clinical trials, not specified	Yuan et al. (2025), Ito et al. (2023)

RNA-based vaccines, comprising mRNA and circular RNA (circRNA) platforms, are emerging as promising therapies for hepatobiliary cancers, particularly HCC (Miao et al., 2021; Yaremenko et al., 2025). In order to overcome the instability of linear mRNA, circRNA-based neoantigen vaccines have been developed, which show enhanced stability and sustained protein expression that promote dendritic cell activation and robust T-cell responses against HCC in preclinical models (Wang F. et al., 2024). MTL-CEBPA is at present the most advanced RNA-based therapeutic analyzed in hepatobiliary malignancies and constitutes a proof-of-concept for LNP-mediated RNA delivery in liver cancers (Sarker et al., 2020). MTL-CEBPA comprises a small activating RNA (saRNA) encapsulated within a SMARTICLES® LNP formulation designed to stimulate the expression of CCAAT/enhancer-binding protein alpha (C/EBPα), a transcription factor with tumor-suppressive and myeloid-modulating functions that is frequently downregulated in HCC. Other RNA approaches, including cancer vaccines and non-coding RNA-based immunotherapies, are under clinical evaluation (Xing et al., 2024; Vianello et al., 2024; Afra et al., 2023).

Recent clinical progress, including the KEYNOTE-942 trial, has demonstrated the potential of combining mRNA vaccines with ICIs, offering new hope for patients with HCC and other difficult-to-treat malignancies (Khattak et al., 2023). In this study, a personalized neoantigen mRNA vaccine (mRNA-4157/V940) was administered in combination with pembrolizumab, a monoclonal immunotherapy drug capable of re-activating the immune system, which resulted in significantly improved recurrence-free survival compared with ICI monotherapy in high-risk melanoma. This highlights the capacity of mRNA platforms to enhance tumor-specific T-cell responses. In the context of HCC, which often surges in an immunologically complex and chronically inflamed liver environment, such combination strategies may help convert immunologically “cold” tumors into “hot” tumors responsive to checkpoint blockade. The identification and characterization of tumor-specific antigens are crucial for the rational design of effective mRNA vaccines, with certain immune subtypes of HCC patients showing particular suitability for vaccination (Fu et al., 2023). Antigens such as such as FXYD6, JAM2, GALNT16, C7, and CCDC146, selected based on tumor-restricted expression, immunogenic potential, and association with clinical outcomes, provide a foundation for developing vaccines capable of eliciting robust and tumor-specific T-cell responses. Interestingly, molecular and immune profiling approaches have revealed distinct immune subtypes among HCC patients, characterized by differing antigen presentation machinery, immune cell infiltration, and checkpoint molecule expression, such as those caused by chronic viral Hepatitis B or C (HBV or HCV respectively) viral infection (De Battista et al., 2024; Ringelhan et al., 2018). Certain immune subtypes, particularly those with preserved antigen presentation capacity and pre-existing immune infiltration, may be especially suitable for mRNA vaccination strategies, as they are more likely to mount effective cytotoxic responses upon antigen stimulation. Other mRNA vaccine strategies include encoding costimulatory molecules such as OX40L to boost T-cell activation and tumor suppression, with promising outcomes in animal models (Deng et al., 2022). By promoting sustained CD4^+^ and CD8^+^ T-cell activation and survival, OX40L-based approaches aim to overcome immune exhaustion and strengthen anti-tumor immunity. Preclinical studies in animal models have demonstrated that mRNA-mediated expression of OX40L can significantly inhibit tumor growth, increase intra-tumoral T-cell infiltration, and synergize with immune checkpoint blockade. These results suggest that mRNA technology can be designed not only to produce tumor antigens but also to deliver immune-stimulating signals that may enhance treatment effectiveness. In hepatobiliary cancers, these strategies may be particularly valuable for counteracting the immunosuppressive liver microenvironment and improving responses to combination immunotherapy, thus warranting further research.

Membrane dynamics play a pivotal role in determining how effectively nanocarriers can deliver mRNA into cells. After systemic administration, nanocarriers face a series of biological hurdles; they must evade rapid clearance, circulate in the bloodstream long enough to reach the target organ, accumulate at the tumor site, and undergo cellular internalization through endocytosis. Subsequently, efficient endosomal escape is required to release mRNA into the cytoplasm, where translation into antigenic proteins occurs (Vishweshwaraiah and Dokholyan, 2022; Liu et al., 2023). LNPs and other advanced nanocarriers are designed to optimize each of these steps.

RNA vaccines are typically delivered using LNPs to enhance stability and promote efficient uptake by dendritic cells, which can induce CD4^+^ and CD8^+^ T cell responses, and have been associated with tumor growth inhibition in preclinical models (Deng et al., 2022; Liu et al., 2023). Neoantigen mRNA vaccines, personalized based on the patient’s tumor mutations, have shown encouraging signals in early clinical and preclinical studies by improving immune recognition and destruction of HCC cells, often in combination with ICIs to overcome the immunosuppressive TME (Han et al., 2023; Tojjari et al., 2023). Modulation of the liver TME, for example, through bile acid metabolism, has been proposed to further enhance mRNA vaccine efficacy by restoring T cell function and reducing immunosuppression (Wang et al., 2025). Clinical trials to evaluate safety and efficacy are ongoing, with preliminary findings suggesting potential for mRNA vaccines as a targeted, personalized, and less toxic therapeutic option for HCC (Lorentzen et al., 2022; Fu et al., 2025). In HCC models, circRNA vaccines are also delivered via LNPs to ensure efficient *in vivo* expression and immune activation (Wang F. et al., 2024). This emerging strategy remains primarily in preclinical stages but may help address some limitations of mRNA vaccines and enhance cancer immunotherapy efficacy. Overall, RNA-based therapies represent a rapidly evolving field with several candidates progressing through clinical evaluation for HCC treatment, supported by strong mechanistic rationale and early evidence of therapeutic benefit. This approach also holds potential for CCA treatment.

## 4 Lipid nanoparticles

Lipid-based nanoparticles, represent a major subclass of nanomaterials alongside polymeric and inorganic nanoparticles. To date, they constitute one of the most innovative and versatile strategies for the delivery of diverse therapeutic and diagnostic molecules, offering customizable platforms for precision medicine applications (Mitchell et al., 2021) (Cheng et al., 2025; Eygeris et al., 2022). Notably, LNPs have been specifically engineered to address the key challenges associated with RNA therapeutics, described below.

### 4.1 LNP structure and cargos

LNPs have long been established as effective delivery systems for an array of cargos. First introduced in 1965, LNP formulations have progressively evolved into clinically relevant nanomedicine platforms, with successive structural refinements improving stability and performance, including the development of nanostructured lipid carriers with enhanced targeting capabilities (Hou et al., 2021). The success of this technology is largely attributed to the physicochemical properties of its lipid components, which are classified based on charge, structure, and complexity into cationic lipids, ionizable lipids, cholesterol, and functionalized lipids (e.g., polyethelene glycol (PEG)-lipids) (Fang et al., 2022). Their structure enables efficient encapsulation of large RNA molecules, such as full-length mRNA, thereby overcoming key limitations of the phospholipid bilayer (Eygeris et al., 2022). These include poor permeability to large, negatively charged molecules and susceptibility to enzymatic degradation. This capability, at therapeutically relevant doses, has been critical for the success of mRNA vaccines and emerging cancer immunotherapies. The high loading efficiency is largely driven by electrostatic interactions between ionizable lipids and negatively charged RNA, enabling stable and reproducible formulation (Eygeris et al., 2022; Fang et al., 2022). However, cargo capacity is not unlimited: augmenting RNA payload can affect particle stability, size distribution, and biodistribution, potentially leading to altered pharmacokinetics or increased off-target accumulation. As a consequence, LNP design requires careful balancing between payload size, delivery efficiency, and safety (Pial et al., 2026).

Beyond mRNA, LNPs can be adapted to deliver smaller regulatory RNAs, such as siRNAs and miRNAs, enabling targeted gene silencing and modulation of oncogenic pathways in hepatobiliary cancers. The modular composition of LNPs allows tuning of delivery properties depending on the cargo, including optimization of encapsulation efficiency, intracellular release, and immune activation.

### 4.2 LNP biology

**FIGURE 1:**
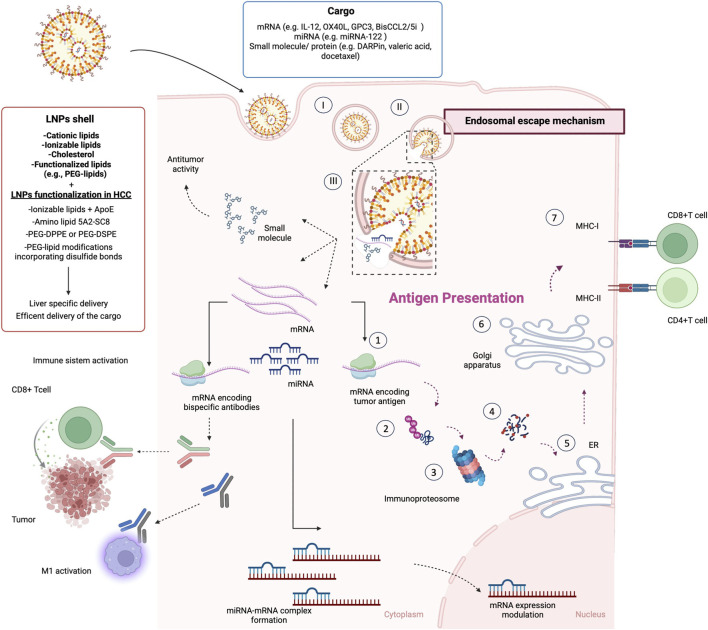
Schematic representation of lipid nanoparticles (LNPs) in hepatocellular carcinoma (HCC) as a delivery platform. The cargo may consist of various molecules, such as mRNA, miRNA, or small molecules/proteins. The LNP shell is composed of different lipids that can be engineered to enhance nanoparticle stability and delivery efficiency. The upper part illustrates the proposed mechanism of endosomal escape: (I) internalization of LNPs through an endocytic mechanism; (II) interactions between ionizable lipids and naturally occurring anionic phospholipids in the endosomal membrane. The acidic endosomal environment facilitates protonation of ionizable lipids into cationic lipids, leading to the formation of ion pairs with anionic lipids; (III) the formation of these ion pairs promotes membrane fusion and disruption, allowing mRNA and other cargo to escape from endosomes. The lower part of the figure represents the process of antigen presentation following LNP delivery. This process begins with (1) translation of mRNA encoding the tumor antigen protein, followed by (2) ubiquitination and (3) proteasomal degradation into peptides, which are subsequently presented (5–6) on the cell surface by major histocompatibility complex (MHC) class I and II molecules, thereby (7) activating CD8^+^ and CD4^+^ T cell–mediated immune responses. miRNA cargo, once released into the cytoplasm, can inhibit the translation of specific target mRNAs. mRNA not encoding antigens can be translated into functional proteins, such as antibodies or cytokines. Small molecules can also be delivered into target cells, where they exert antitumor activity.

LNPs are currently the most established platform for mRNA delivery, owing to their ability to efficiently encapsulate and protect mRNA, enhance cellular internalization, and promote endosomal escape (Eygeris et al., 2022; Hou et al., 2021; Alshehry et al., 2025). Delivery of mRNA via LNPs promotes physiological cellular uptake through endocytosis. Ionizable lipids play a crucial role in facilitating endosomal escape, preventing cargo degradation in lysosomes (Figure 1). This is essential to preserve the therapeutic activity of mRNA and the gene-silencing efficacy of siRNA and miRNA cargo (Varkouhi et al., 2011; Semple et al., 2010). This class of lipids is specifically engineered to acquire a cationic charge under acidic conditions, enabling a more efficient release and translation of mRNA, subsequently processed by the immunoproteasome (Wittrup et al., 2015).

The high biocompatibility and clinical validation of LNPs are exemplified by the successful deployment of mRNA-based COVID-19 vaccines. In this context, several studies have demonstrated that both the LNP components and the mRNA itself contribute to the adjuvant effect, promoting robust humoral and cellular immune responses. These components specifically play a key role in the antigen presentation by antigen-presenting cells (APCs) via major histocompatibility complex class I (MHC-I) molecules that drives CD8^+^ T-cell activation, whereas presentation through MHC class II (MHC-II) molecules stimulates CD4^+^ T-cell responses, ultimately supporting B-cell activation and antibody production (Cagigi and Loré, 2021). Moreover, lipid components can be engineered to minimize plasma protein adsorption, thereby prolonging systemic circulation and improving tissue targeting; however, synthetic lipid-based systems may still trigger immune responses and exhibit preferential accumulation in off-target organs such as the liver (Nawaz et al., 2025).

### 4.3 LNP in hepatobiliary cancers therapy

Since the approval of COVID-19 mRNA vaccines, LNP–mRNA combinations have demonstrated clinical utility in vaccination and shown promise across multiple therapeutic areas. In this review, we discuss the potential of this technology in hepatobiliary malignancies. In hepatic diseases, including genetic and metabolic disorders, infections, and malignancies, LNPs have been intensively investigated for nucleic acid delivery in gene editing, enzyme replacement therapies, and gene silencing approaches targeting viral infections or hyperlipidemia. Moreover, LNP-based strategies have been evaluated in preclinical and early clinical studies across several malignancies, with clinical trials reported in cancers such as melanoma and pancreatic cancer (Rojas et al., 2023; Weber et al., 2024).

Following the success of LNP-based mRNA vaccines, efforts have focused on improving LNP composition. Structural modifications can improve biological performance and expand functional versatility. In particular, altering the structure of ionizable lipids represents a promising strategy to improve LNP efficacy. Akinc et al. demonstrated that apolipoprotein E (ApoE), a key mediator of clearance and hepatocellular uptake, can associate with ionizable lipids, resulting in efficient siRNA delivery; this interaction was less pronounced with cationic lipids (Akinc et al., 2010). Based on this observation, libraries of ionizable lipids with improved *in vivo* delivery properties were generated. Among these, the amino lipid 5A2-SC8 was reported to exhibit high ApoE adsorption, which may facilitate hepatocyte uptake *v*ia the LDL receptor (Johnson et al., 2022). In addition, modifications of the PEG shield were found to influence LNP uptake. LNPs containing PEG-DPPE or PEG-DSPE displayed low uptake *in vitro* but achieved measurable tumor delivery *in vivo*, whereas PEG-DMPE-containing LNPs showed the opposite trend, highlighting the potential importance of appropriate PEG-lipid selection (Li et al., 2013). Furthermore, PEG-lipid modifications incorporating disulfide bonds combined with transferrin insertion, were reported to prolong circulation time *in vivo*. Zhao et al. applied this strategy in an *in vivo* HCC model, demonstrating increased tumor accumulation and improved cargo release in tumor-bearing mice (Zhao et al., 2019).

Over the past decade, LNPs have also assumed a major role in immunotherapy and immune system modulation. Activation of CD4^+^ and CD8^+^ T cells is a key requirement for effective tumor recognition and eradication. In HCC, LNPs delivering mRNA encoding immunomodulatory targets have demonstrated the feasibility of mRNA-based vaccination strategies. More broadly, these approaches illustrate how LNPs can be tailored to different RNA modalities, ranging from antigen-encoding mRNA for immune activation to regulatory RNAs that modulate the TME and counteract tumor-driven immune and therapeutic resistance mechanisms. Deng et al. investigated an optimized low-immunogenicity OX40L mRNA vaccine combined with miRNA-122 in HCC models. OX40L acted as a costimulatory molecule, enhancing IL-2 expression *in vitro*. Intratumoral delivery *in vivo* resulted in CD4^+^CD69^+^ and CD8^+^CD69^+^ T cell infiltration and activation, supporting therapeutic efficacy (Deng et al., 2022). In a separate study, tumor-derived RNA encapsulated in LNPs was used to target dendritic cells, inducing a potent T cell response. *In vivo* experiments demonstrated effective prevention and inhibition of HCC growth (Zhang et al., 2021).

Another immunotherapeutic strategy involves bispecific antibodies, engineered molecules that can bind to two different targets at the same time. While one part binds to tumor cells, the other recruits T cells, effectively bringing the immune system directly into contact with cancer and triggering tumor cell killing. Huang et al. explored this concept using an mRNA-LNP encoding a glypican-3 (GPC3, a protein commonly found in HCC)-targeting T cell engager in HCC, showing strong antitumor activity. This formulation induced the presence of CD8^+^ T cells within tumors, both early-stage precursors and more mature memory cells that are crucial for long-term immune protection. These encouraging results have led to an ongoing, first-in-human clinical trial to evaluate safety and early efficacy (NCT06689540, www.clinicaltrial.gov, last accessed on 20 January 2026) (Huang Y. et al., 2025).

A related strategy uses a bispecific inhibitor BisCCL2/5i, which binds and neutralizes two signaling molecules CCL2 and CCL5, which attract immunosuppressive cells. When encapsulated in a clinically approved LNP platform, mRNA encoding BisCCL2/5i naturally accumulated in the liver, leading to macrophages’ polarization toward an M1 phenotype, a more inflammatory and tumor-fighting state. This reduced immunosuppression within the TME. When this approach was combined with PD-L1 blockade, it led to long-term survival in mouse models of primary liver cancer and liver metastases from colorectal and pancreatic cancers (Wang et al., 2021).

Another emerging approach involves delivery of IL-12 mRNA using LNPs, particularly in MYC-driven HCC. *In vivo* studies showed that IL-12-LNPs induced increased infiltration of activated CD44^+^CD3^+^CD4^+^ T helper cells into tumors and higher interferon-γ production (Lai et al., 2018).

Beyond nucleic acid delivery, lipid nanoparticles have been applied to the delivery of non-nucleic acid therapeutics. Unlike nucleic acids, which are negatively charged and readily associate with positively charged lipids, efficient delivery of small-molecule drugs often requires lipid modification. Haley et al. demonstrated that particle modifications enabled targeting of mutant RAS proteins, which are typically considered undruggable due to poor cytosolic accessibility. Delivery of a charge-modified designed ankyrin repeat protein (DARPin, a small engineered binding protein derived from ankyrin repeat motifs) targeting RAS, engineered with D30, was associated with enhanced intracellular delivery. Both *in vitro* and *in vivo* studies showed antitumor activity compared to free protein controls (Haley et al., 2023). In addition, LNP-mediated delivery of valeric acid, a short-chain fatty acid, demonstrated antitumor effects and improved survival. Valeric acid exhibited potential histone deacetylase inhibitory activity, as shown by structural target prediction and enzymatic assays (Han et al., 2020).

The incoportation of long-chain fatty acids and triglycerides enables the generation of solid lipid nanoparticles (sLNPs). The rigidity of the lipid core represents a critical factor governing drug release kinetics. In HCC models, sLNPs encapsulating docetaxel have demonstrated enhanced antitumor efficacy while maintaining a favorable safety profile, with no evident systemic toxicity in the treatment of locally advanced and metastatic disease (Xu et al., 2009). Overall, these non–nucleic-acid-based strategies further broaden the therapeutic potential of LNPs in cancer treatment.

Interestingly, nanotechnology has revolutionized HCC treatment by enabling nanoparticle-based delivery systems that can modulate the immunosuppressive TME, improve drug bioavailability, and overcome biological barriers, with some formulations progressing toward clinical application (Mi et al., 2025; Aldhubiab et al., 2025). RNA nanotherapeutics using lipid and polymer nanoparticles have shown considerable promise in preclinical and early clinical stages for HCC by targeting molecular pathways and the TME, though clinical translation is ongoing (Figure 1) (Yuan et al., 2025). Several nanocarrier types, including liposomal and polymer-based systems, have been approved by the FDA or are currently under clinical investigation for cancer therapy broadly, but active targeting nanocarriers specifically for hepatobiliary cancers are still largely confined to preclinical or early-phase clinical evaluation (Aldhubiab et al., 2025; Attia et al., 2019). Challenges such as rapid clearance by the immune system, TME heterogeneity, and manufacturing scale-up continue to limit clinical translation. Overall, while preclinical and some early clinical studies support the potential of nanocarriers to overcome TME barriers in hepatobiliary cancers, further clinical trials are required to define their safety, efficacy, and optimized delivery strategies in patients.

Building on these promising results in HCC, LNP-mRNA delivery strategy may represent a powerful platform to translate its efficacy to CCA. However, cancer immunotherapy has shown limited efficacy in CCA across both conventional and novel treatments, including immune checkpoint blockade. Likewise, LNP-mRNA could offer a valid immunotherapy approach; however, its application is currently constrained by the limited integration of vaccine design and patient selection (Tang et al., 2022). Huang et al. performed an integrative bioinformatic analysis of TCGA and GEO datasets to identify candidate tumor antigens suitable for mRNA vaccine development. By analysing amplified and mutated genes and correlating them with survival outcomes, three antigens, CD247, FCGR1A, and TRRAP, were identified (Huang et al., 2021). High expression of these genes was found to be associated with improved overall and recurrence-free survival and positively correlated with infiltration of antigen-presenting cells, suggesting potential immunogenic relevance. Furthermore, immune profiling identified two CCA subtypes, with the immune “cold” subtype (IS2) proposed as a potential target population for vaccination. These findings provide a preliminary framework for antigen selection and patient stratification in future CCA mRNA vaccine strategies (Huang et al., 2021). In spite of these promising insights, further studies are needed to better define immunogenic antigens and the onco-immunological landscape of CCA thereby supporting the rational clinical development of CCA mRNA vaccine approaches in this setting (Tang et al., 2022).

## 5 Extracellular vesicles

In contrast to synthetic nanocarriers, EVs are nanoscale, naturally occurring, cell-derived particles that are intrinsically involved in the physiological transfer of bioactive molecules within the body. Their role in intercellular communication renders them particularly well-suited for delivering regulatory RNA molecules that modulate gene expression and cellular signaling in recipient cells. Due to their intrinsic biocompatibility, ability to trigger minimal immune responses and capacity for selective cellular targeting, EVs are increasingly being explored across various cancers as versatile platforms for delivery of therapeutic payloads, thus offering a promising ground for use in hepatobiliary diseases (Yu et al., 2023; Abels and Breakefield, 2016).

### 5.1 EV biology

EVs is the umbrella term describing mainly vesicles formed through two pathways: exosomes originate inside the cell, where inward budding of endosomal membranes gives rise to vesicles; microvesicles form directly at the cell surface through outward budding of the plasma membrane (Théry et al., 2018). Thus, the cargo carried by EVs is not random. Once released, EVs travel through extracellular fluids and can be taken up by other cells through several mechanisms, including receptor-mediated endocytosis, phagocytosis, macropinocytosis, or direct membrane fusion. Through these mechanisms, EVs can functionally transfer their cargo to recipient cells, leading to measurable changes in gene expression, signaling pathways, and cellular behavior (Chrzanowski and Wolfram, 2026). This ability of EVs is increasingly being exploited for therapeutic purposes. Molecules on the EV surface, particularly specific proteins and lipids, help determine which cells they interact with and how they are processed once inside the cells (Yu et al., 2023; Ginini et al., 2022).

The contents of EVs are remarkably diverse. They can carry proteins, various forms of RNA (including mRNA, miRNA, and lncRNA), DNA, and small metabolites, and in some cases, even organelles (Ferro et al., 2024; Payandeh et al., 2024). This cargo allows EVs to influence gene expression, signaling pathways, and overall cellular behavior in recipient cells (O'Brien et al., 2020; Gregory and Rimmer, 2023). EVs often mirrors the identity and condition of the originating cell, including its signaling activity, metabolic state, and whether it is under stress or affected by disease, making them ideal tool for biomarker development (Dixson et al., 2023). In this way, EVs serve as both messengers and modulators within biological systems.

### 5.2 EVs in tumor progression

**FIGURE 2:**
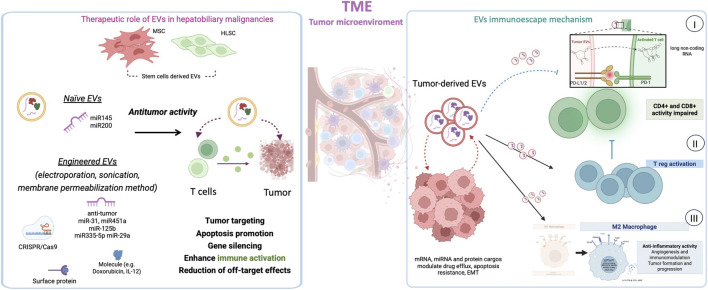
Schematic representation of the roles of extracellular vesicles (EVs) in the hepatobiliary tumor microenvironment (TME). On the left side of the figure, the therapeutic potential of EVs derived from naïve and engineered stem cells, such as mesenchymal stem cells (MSCs) and human liver stem cells (HLSCs), is illustrated. On the right side, the functions of EVs released within the TME are depicted, highlighting their autocrine effects on tumor cells and, in particular, their impact on the immune system. These effects occur through: (I) direct interactions between tumor-derived EVs and immune cells, including modulation of the PD-1/PD-L1 axis and delivery of long non-coding RNAs; (II) modulation of the immune response via activation of regulatory T cells (Tregs), leading to suppression of CD8^+^ T cells; and (III) promotion of M2 macrophage polarization, resulting in an anti-inflammatory phenotype.

EVs derived from stem cells show significant anti-tumor properties and are attracting increasing attention as potential tools for hepatobiliary cancers, including HCC. EVs from human liver stem cells (HLSCs) have demonstrated measurable anti-tumor effects in experimental models. These vesicles have been shown potential to inhibit cancer stem cell (CSC)-derived tumor growth through multiple coordinated mechanisms: promoting apoptosis, reducing proliferation and invasion, and interfering with tumor vascularization, partly through the transfer of specific antitumor miRNAs such as miR-145 and miR-200 family members, known to regulate pathways involved in stemness, EMT, and survival (Figure 2) (Brossa et al., 2020; Zhou M. et al., 2024). The next step will be to fully clarify the efficiency and selectivity of miRNA delivery in patients. Mesenchymal stem cell (MSC)-derived EVs present a more complex picture. Depending on context, they have been reported to exert instead demonstrate dual roles in cancer, both pro- and anti-tumor effects, which has prompted a shift toward engineering these vesicles to enhance their targeting specificity and deliver therapeutic cargo like siRNAs or chemotherapeutic drugs, while improving anti-tumor efficacy while minimizing off-target toxicity (Weng et al., 2021; Hwang et al., 2025). For instance, desialylated MSC-EVs loaded with doxorubicin show enhanced targeting and inhibition of HCC cells via recognition by hepatoma-specific receptors, leading to improved drug uptake and tumor suppression *in vivo* (Yang et al., 2022). Moreover, engineered EVs can overcome drug resistance in HCC by delivering CRISPR/Cas9 systems targeting key genes involved in cancer stemness and sorafenib resistance; this modification has led to a reduction in CSC populations and enhancement of treatment response (He C. et al., 2023). However, due to the complexity of gene editing in clinical contexts, issues such as delivery efficiency, off-target effects, and long-term safety must be meticulously assessed to translate these findings into reliable clinical therapies.

On the other hand, the same biological features that render EVs attractive as delivery tools also underpin their role in disease progression. Their small size and membrane composition allow EVs to penetrate fibrotic and hypoxic tumor regions, leading to modulation of the TME, chemoresistance, and immune evasion (Figure 2). Tumor-derived EVs carry bioactive molecules such as non-coding RNAs, proteins, and lipids that remodel the TME by promoting angiogenesis, extracellular matrix remodeling, and EMT, which support tumor growth, invasion, and metastasis (Augello et al., 2024; Pascut et al., 2020; Tao and Guo, 2020). A particularly challenging aspect is their contribution to chemoresistance. EVs can transfer miRNAs and other cargo that regulate drug efflux, apoptosis resistance, and EMT (Figure 2), thereby reducing the efficacy of chemotherapy and targeted therapies (Zelli et al., 2022; Mir et al., 2024). The cargo can be delivered to cancer cells, stromal fibroblasts, endothelial cells and immune cells.

EVs also play a substantial role in immune evasion by delivering immunosuppressive molecules that impair T cell activation and promote regulatory T cell (Treg) expansion, which suppresses effective antitumor immunity (Liu et al., 2025; Chen et al., 2023a). Tumor-derived EVs carry immune checkpoint proteins such as Programmed Death-Ligand 1 (PD-L1), which bind to programmed cell death protein 1 (PD-1) receptors on T cells, causing T cell exhaustion and reduced cytotoxic activity, thereby facilitating tumor escape from immune surveillance and resistance to immune checkpoint blockade therapies (Zhao et al., 2024; Raimondo et al., 2020). Beyond T cells, EVs influence other immune components. It has been shown that EVs can reduce natural killer (NK) cell cytotoxicity and polarize macrophages toward immunosuppressive M2 phenotypes, further dampening antitumor immune responses within the TME (Liu et al., 2025; Kuang et al., 2025). Specific cargoes are also emerging: for example, EV-associated lysyl oxidase like-4 (LOXL4) can activate signaling axes like the STAT1/PD-L1 axis in macrophages, enhancing immunosuppression and inhibiting CD8^+^ T cell killing of HCC cells (Zhao et al., 2024). Similarly, EVs can transport long noncoding RNAs such as HDAC2-AS2 that suppress CD8^+^ T cell cytotoxicity by interfering with key transcription factors, contributing to immune exhaustion and poor prognosis in HBV-associated HCC (Gao et al., 2025). Taken together, these mechanisms collectively create a broadly immunosuppressive milieu that hinders effective immune responses and poses a major challenge for immunotherapy (Figure 2), making EV-mediated immune suppression a promising target to improve treatment outcomes in cancer (Chen K. et al., 2024; Nguyen et al., 2022). The dual nature of EVs, both as potential therapeutic carriers and as mediators of disease, indicates how the cellular source of EVs is critical. EVs derived from different cell types within the TME carry distinct molecular signatures, and these differences can significantly affect cancer progression, drug resistance, and immune modulation (Su et al., 2024). A more precise understanding of these variables will be essential if EVs are to be safely and effectively harnessed for RNA-based therapies in hepatobiliary cancers.

### 5.3 EVs in therapy

Therapeutically, EVs have emerged as versatile delivery platforms capable of transporting chemotherapeutics, nucleic acids, immunomodulators, and gene-editing tools with high precision, although their clinical translation is still under active investigation. In the context of RNA therapeutics, EVs are particularly suited for the delivery of small regulatory RNAs (Figure 2), such as miRNAs and siRNAs, which they naturally encapsulate and protect from degradation, while facilitating cellular uptake (Chen et al., 2023b). This makes them attractive platforms for modulating oncogenic pathways, reversing drug resistance, and reshaping the tumor microenvironment, to be explored in hepatobiliary cancers.

EVs can also be engineered to carry therapeutic RNA, including vaccine-related cargo. EVs are typically engineered either by modifying the parent cells or by directly altering isolated vesicles. In cell-based approaches, donor cells are genetically engineered to express specific proteins, RNA cargos, or targeting ligands that are be selectively incorporated into EVs during biogenesis (Wang et al., 2022; Cecchin et al., 2023). Alternatively, cellular preconditioning (such as hypoxia or cytokine exposure) may alter EV biogenesis and cargo composition, potentially increasing the relative abundance of specific functional molecules within EV cargo (Kalluri and LeBleu, 2020). On the other hand, in cell-free approaches, isolated EVs are loaded therapeutic RNAs, drugs, or proteins through physical or chemical methods such as electroporation, sonication, or membrane permeabilization. Surface engineering techniques, such as click chemistry, offer strategies to enhance targeting specificity toward selected tissues or cell types (Hu et al., 2025). By enabling the precise functionalization of surfaces with targeting moieties, these methods can improve selective recognition and uptake by desired biological targets.

Engineering strategies, including cargo loading with small RNAs or mRNA, surface modification with tumor-specific ligands or nanobodies, and incorporation of immunostimulatory molecules such as IL-12, enhance tumor selectivity, immune activation, and therapeutic outcomes (Dong et al., 2023; Wu et al., 2025; Zhang et al., 2025). Due to their intrinsic liver tropism, EVs are particularly attractive for hepatobiliary malignancies (Xu et al., 2025). EVs derived from activated immune cells or engineered for catalytic tumor cell killing combine therapeutic and imaging functionalities, representing potential versatile theranostic platforms for precision hepatobiliary cancer therapy. Engineered EVs displaying pathogen proteins or loaded with mRNA may help overcome several limitations of LNP-based mRNA vaccines, such as liver accumulation and toxicity, by enhancing tissue-specific delivery and immune activation (Zhang B. et al., 2024; Li et al., 2025). Compared with cell-based vaccine platforms, EV-based vaccines can induce broader and more effective immune responses against tumor antigens, with advantages including high safety, ease of preservation, and superior tissue delivery (Meng et al., 2025). In HCC, EVs have been successfully used to deliver antitumor miRNAs, suggesting their potential for mRNA vaccine delivery as well (Pomatto et al., 2019; Zhou et al., 2022). Multiple studies demonstrate that EV-mediated miRNA delivery can promote apoptosis, silence oncogenic pathways and dampen tumor growth. Examples include miR-31 and miR-451a, which when loaded into plasma-derived EVs induce apoptosis by targeting anti-apoptotic pathways in HepG2 cells (Pomatto et al., 2019). MiR-125b delivered *via* EVs from genetically modified MSCs inhibits HCC cell proliferation by modulating targets in the p53 signaling pathway (Baldari et al., 2019). MiR-335-5p loaded into stellate cell-derived EVs suppresses HCC cell proliferation and invasion, inducing tumor shrinkage by downregulating specific mRNA targets (Wang et al., 2018). In addition, miR-29a delivered by EVs blocks autophagy and induces apoptosis in HCC cell lines, reducing proliferation and colony formation (Seydi et al., 2024). These findings show the potential of EV-mediated delivery of various miRNAs as therapeutic strategies against HCC. While clinical translation is still in early stages, EV-based RNA vaccines represent a novel and promising immunotherapy strategy for hepatobiliary cancers, potentially improving efficacy and safety over current RNA vaccine platforms (Li et al., 2025; Yao et al., 2024). Collectively, these strategies allow EVs to be tailored to improve tumor targeting, immune activation, and antitumor efficacy while minimizing off-target toxicity.

Compared to LNPs, EV-based delivery systems still face challenges regarding loading efficiency, scalability, and control over transgene expression (Kirian et al., 2024). For this reason, EVs are often viewed as complementary rather than alternative platforms, offering enhanced biological targeting and biocompatibility. Moreover, unlike synthetic systems, the cargo capacity of EVs is more constrained and less predictable, reflecting their biological origin. Although EVs naturally carry a diverse range of biomolecules, including small RNAs, proteins, and lipids, efficient loading of exogenous therapeutic cargo, in particular large molecules such as mRNA, remains technically challenging. Current approaches, such as electroporation, transfection of donor cells, or membrane permeabilization, often result in variable encapsulation efficiency and may compromise cargo integrity (Oshchepkova et al., 2023). Thus, EVs are currently considered particularly well suited for the delivery of smaller regulatory RNAs, such as miRNAs and siRNAs, which align with their physiological function. Despite these limitations, ongoing advances in EV engineering continue to improve loading strategies and may broaden their capacity for more complex therapeutic cargos.

Overall, LNPs and EVs offer complementary strengths as RNA delivery platforms. While LNPs excel in the efficient, scalable delivery of synthetic RNA constructs, particularly mRNA, EVs provide a biologically adapted system for the transfer of regulatory RNAs within complex tumor microenvironments (Hou et al., 2021; Prieto-Vila M and Ochiya, 2021). These distinct properties highlight the importance of selecting, or potentially combining, delivery platforms based on the specific RNA cargo and therapeutic goal. From a delivery perspective, LNPs offer higher and more controllable cargo loading, particularly for large RNA constructs (Cullis PR, 2024), whereas EVs provide a biologically refined but more capacity-limited system, naturally suited for the delivery of smaller regulatory molecules particularly to the liver due to their intrinsic biodistribution (Wiklander et al., 2015). These differences in cargo capacity further support the development of hybrid systems, designed to combine the loading efficiency of LNPs with the targeting capabilities of EVs.

### 5.4 EVs as biomarkers

Beyond their role as delivery vehicles, EVs are emerging as powerful diagnostic and prognostic biomarkers. Their cargo reflects the TME and disease state, enabling early, minimally invasive detection of HCC and monitoring of therapeutic responses (Juratli et al., 2024; Liu et al., 2024). Recent advances in EV capture and analysis platforms, including EV Click Chip or the tidal microfluidic chip, allow sensitive and specific molecular profiling of HCC-derived EVs through the quantification of tumor-associated mRNA and protein markers, supporting early diagnosis and treatment monitoring (Sun et al., 2020; Yi et al., 2025). In parallel, digital scoring systems that quantify tumor-derived EV molecular cargo are emerging as highly accurate tools for early detection and clinical monitoring. These platforms integrate EV enrichment with high-resolution molecular assays to compute composite scores reflecting the tumor burden and biological activity. One example is the HCC EV TR Score (Hepatocellular Carcinoma Extracellular Vesicle Treatment Response Score), which is derived from a digital scoring assay and combines click chemistry-mediated capture of HCC-specific EVs with reverse transcription digital polymerase chain reaction (RT-dPCR) quantification of EV-associated transcripts to monitor therapeutic response (Zhao et al., 2025). In a retrospective study, the HCC EV TR Score distinguished viable from nonviable tumors after locoregional or surgical therapy with high sensitivity and specificity, and detected residual or recurrent disease earlier than magnetic resonance imaging (MRI) alone, higlighting its prognostic value. Thus, these approaches suggest that EV-based digital biomarkers may complement imaging and serum markers by providing a quantitative, minimally invasive measure of tumor viability.

## 6 Biodistribution and theranostics

Imaging techniques play a central role in nanocarrier research by enabling visualization, tracking, and quantification of biodistribution, drug release, and interactions within biological systems. Advanced methods such as Raman imaging, including coherent anti-Stokes Raman scattering (CARS) and stimulated Raman scattering (SRS), provide label-free, non-invasive imaging of nanocarriers at the single-cell level, offering insight into *in vivo* distribution and mechanisms of action (Vanden-Hehir et al., 2019; Gao et al., 2024). Fluorescence imaging, especially in the near-infrared (NIR-II) window, enables real-time, high-resolution tracking of nanocarriers and their pharmacokinetics, while aggregation-caused quenching (ACQ) probes can improve imaging accuracy by minimizing background interference (Zhang Z. et al., 2024). Other powerful modalities include mass spectrometry imaging for label-free visualization of *in situ* drug release at the tissue and sub-organ level, and multimodal imaging platforms that integrate fluorescence, MRI, and PET for comprehensive biodistribution and safety assessment (Xue et al., 2018; Zhang et al., 2020; Szczęch et al., 2020). Complementing these approaches, microscopy techniques such as confocal laser scanning microscopy (CLSM), scanning and transmission electron microscopy (SEM/TEM), and atomic force microscopy (AFM) remain essential for characterizing nanocarrier morphology, size, and surface properties (Falsafi et al., 2020).

*In vivo* imaging EVs and LNPs is essential for understanding their biodistribution, pharmacokinetics, and therapeutic behavior. Recent advances in live and high-resolution microscopy, together with innovative labeling strategies such as bioluminescence, fluorescence, magnetic resonance, and nuclear imaging, have enabled visualization of EVs at the single-vesicle level within physiological environments (Verweij et al., 2021; Arifin et al., 2022; Gupta et al., 2020). Multiplexed approaches, including barcoded hybrids of EVs and LNPs, allow simultaneous tracking of multiple EV populations *in vivo*, revealing tissue-specific tropism and delivery capabilities. For example, Ivanova et al. mapped the tropism of HAP1-derived EV hybrids from 16 different cell sources, and showed their preferential targeting of the lungs in a mouse model (Ivanova et al., 2025). Studies in zebrafish and rodents have provided further insights into EV release, uptake, and clearance dynamics, highlighting rapid organ distribution post-administration and the impact of injection routes on biodistribution (Gupta et al., 2020; Verdi et al., 2021). Overall, these imaging platforms are accelerating the clinical translation of EV- and LNP-based therapeutics by enabling real-time, non-invasive tracking and functional assessment in living systems (Gangadaran et al., 2024; Rakshit and Pal, 2024; Gao et al., 2021). However, challenges remain in achieving highly specific, sensitive, and biocompatible labeling methods that accurately reflect EV behavior without altering their nanocarrier function or biodistribution (Chuo et al., 2018; Li et al., 2020). Continued improvements in imaging technologies will be important for advancing nanocarrier-based theranostics in hepatobiliary cancers (Vanden-Hehir et al., 2019; Bose et al., 2018; Farokhi et al., 2019).

## 7 Clinical applications

### 7.1 LNPs mRNA-based strategy in HCC and biliary tumors

The COVID-19 pandemic has markedly accelerated global interest in LNP mRNA-formulated vaccines. Indeed, the LNP-mRNA platforms represent the most clinical advanced nucleic acid-based delivery systems explored as therapeutic strategy in oncological preclinical and clinical trials (Yaremenko et al., 2025). Firstly, due to an improved technological advancement, LNP-mRNA-based strategies are fast, inexpensive from an economical point of view and well tolerated, easily degraded and do not integrate into the host genome (Guan and Rosenecker, 2017). Moreover, mRNA vaccines can elicit both humoral and cell-mediated immune responses (Lorentzen et al., 2022). As discussed throughout this review, mRNA-based cancer vaccines are designed to initiate or amplify effective antitumor immunity by enabling antigen-presenting cells to process and present tumor-associated antigens via both MHC class I and class II pathways. This dual presentation promotes the activation of cytotoxic CD8^+^ T cells as well as helper CD4^+^ T cells, which are essential for sustaining and shaping antitumor immune responses (Shi et al., 2024; Qiu et al., 2023; Castaño et al., 2025).

Therefore, mRNA technology has emerged as a promising strategy in cancer, which faces an important global health burden. As of 2026, up to 130 clinical trials have been registered on ClinicalTrials.gov evaluating the mRNA efficacy in patients with malignancies, providing clinical evidences and generating milestone data of their effectiveness and safety. To date, the predominant strategy for mRNA cancer vaccine development has focused on personalized formulations encoding patient-specific neoantigens and tumor-associated antigens, with the aim of eliciting robust activation of the host immune system to selectively eliminate malignant cells (Rojas et al., 2023; Killock, 2024). By leveraging endogenous antigen presentation pathways, these vaccines are designed to elicit potent and durable antitumor immune responses. mRNA-based immunotherapies can be administered as monotherapy or integrated with complementary treatment modalities, including immune checkpoint blockade and surgical tumor resection (Rojas et al., 2023; Weber et al., 2024). Collectively, this approach represents a highly adaptable and evolving therapeutic paradigm, with considerable potential to improve clinical outcomes in cancers that are refractory to conventional treatments.

In the context of personalized vaccine, mRNA-4157 (V940) exemplifies this approach by integrating tumor whole-exome and transcriptome sequencing with HLA typing to computationally identify and encode up to 34 patient-specific neoantigens within a single optimized mRNA molecule formulated in LNPs (Weber et al., 2024). Endogenous expression of these neoantigens enables physiological antigen processing and presentation, hence activating both CD8^+^ and CD4^+^ T-cell responses. This process may also promote epitope spreading, thereby broadening antitumor immunity and limiting immune escape. In the randomized phase 2b KEYNOTE-942 trial, mRNA-4157 combined with PD-1 blockade improved recurrence-free and distant metastasis-free survival compared with pembrolizumab alone, with a favorable safety profile.

In another clinical study, proof-of-concept for personalized neoantigen mRNA vaccination in pancreatic ductal adenocarcinoma (PDAC), a malignancy largely refractory to conventional immunotherapy, has been demonstrated (Rojas et al., 2023). Patient-specific somatic mutations were identified by tumor–normal sequencing and used to generate individualized RNA vaccines encoding multiple predicted neoantigens (BNT122). Despite the low mutational burden and immunosuppressive microenvironment of PDAC, vaccination elicited robust, polyfunctional CD4^+^ and CD8^+^ T-cell responses against vaccine-encoded neoantigens in most patients. Vaccine-induced T cells showed functional effector phenotypes, clonal expansion, and persistence, indicating effective *de novo* priming rather than amplification of pre-existing immunity. These findings demonstrate that neoantigen mRNA vaccines can overcome immune ignorance in poorly immunogenic tumors and establish a strong biological rationale for combining personalized mRNA vaccination with checkpoint blockade or other microenvironment-modulating therapies.

Building on these advances, promising efforts are also underway in other solid tumors, including HCC (Table 4). In fact, several mRNA-based vaccine trials are currently in progress for HCC. In this context, trial NCT05761717 is investigating a personalized mRNA-based tumor vaccine combined with sintilimab, a human IgG4 monoclonal antibody targeting PD-1 that functions as an ICI to enhance T-cell–mediated antitumor responses. By encoding multiple tumor-associated antigens, the vaccine is designed to induce a robust, targeted immune response and has shown marked tumor regression and immune enhancement in preclinical models, supporting its potential clinical benefit (Shi et al., 2024). Another clinical investigation (NCT05981066) is assessing the safety of a personalized neoantigen mRNA vaccine in patients with relapsed or refractory HCC. This individualized vaccine strategy employs mRNA constructs encoding tumor-derived neoepitopes unique to each patient, with the aim of eliciting highly selective antitumor immune responses. The study is designed to evaluate tolerability and immunogenicity, potentially establishing a new therapeutic avenue for individuals who have exhausted standard treatment options. Moreover, a phase I clinical trial (NCT05738447) is exploring the therapeutic potential of an mRNA vaccine in patients with advanced HBV-positive HCC (Alrumaihi et al., 2025). By encoding hepatitis B virus (HBV) antigens, this strategy aims to activate immune responses specifically targeting HBV-associated malignant hepatocytes. Preclinical evidence indicates that this approach induces potent and durable immune responses, resulting in long-term protection against HBV-related HCC.

**TABLE 4:** Updated Clinical trial using LNPs-mRNA as therapy for HCC (https://clinicaltrials.gov/).

NCT number	Study Title	Study URL	Study status	Conditions	Interventions	Study type
NCT07077356	Application of mRNA Vaccine in Liver Transplantation for Hepatocellular Carcinoma	https://clinicaltrials.gov/study/ NCT07077356	RECRUITING	HCC	BIOLOGICAL: HBV mRNA vaccine	Interventional
NCT07053072	PD-1 mRNA LNP Vaccine for Advanced Primary Hepatocellular Carcinoma	https://clinicaltrials.gov/study/ NCT07053072	RECRUITING	Liver Cancer	DRUG: Low Dose PD-1 mRNA LNP Vaccine|DRUG: Medium dose PD-1 mRNA LNP vaccines|DRUG: High dose PD-1 mRNA LNP vaccines	Interventional
NCT07341321	SARS-CoV-2 mRNA Vaccination in Patients With Hepatocellular Carcinoma Treated With Immune Checkpoint Inhibitors	https://clinicaltrials.gov/study/ NCT07341321	NOT_YET_RECRUITING	mRNA Vaccination| HCC| Checkpoint Inhibitor	​	Observational
NCT05761717	Clinical Study of mRNA Vaccine in Patients With Liver Cancer After Operation	https://clinicaltrials.gov/study/ NCT05761717	UNKNOWN	Post-operative HCC	DRUG: Neoantigen mRNA Personalised Cancer vaccine in combination with Stintilimab I njection	Interventional
NCT05738447	Application of mRNA Immunotherapy Technology in Hepatitis B Virus-related Refractory Hepatocellular Carcinoma	https://clinicaltrials.gov/study/ NCT05738447	UNKNOWN	Liver Cancer| HCC	BIOLOGICAL: HBV mRNA vaccine	Interventional
NCT07077369	WGc-0201 Plus Tislelizumab in HCC With High Risk of Recurrence and Metastasis After Radical Therapy	https://clinicaltrials.gov/study/ NCT07077369	NOT_YET_RECRUITING	HCC	BIOLOGICAL: WGc-0201 injection|DRUG: Tislelizumab	Interventional
NCT05981066	A Clinical Study of mRNA Vaccine (ABOR2014/IPM511) in Patients With Advanced Hepatocellular Carcinoma	https://clinicaltrials.gov/study/ NCT05981066	UNKNOWN	Advanced HCC	DRUG: Neoantigen vaccine, I.M injection	Interventional

Emerging mechanistic and clinical evidence suggests that SARS-CoV-2 mRNA vaccines may enhance tumor responsiveness to ICIs. Grippin et al. demonstrated that mRNA vaccination induces systemic type I interferon responses, promotes activation of antigen-presenting cells, and increases PD-L1 expression on tumor cells, thereby sensitizing tumors to ICIs in malignancies such as non-small cell lung cancer and melanoma (Grippin et al., 2025). Consistent with these findings, retrospective analyses indicated that receiving a COVID-19 mRNA vaccine within a defined interval (100 days) prior to ICI therapy was associated with improved overall and progression-free survival. However, no such data exist for HCC, which is characterized by an immunosuppressive, “immune-cold” TME limiting antitumor immunity (Hagiwara et al., 2023). Although ICIs now represent a cornerstone of systemic HCC treatment, clinical responses remain heterogeneous. Given the widespread administration of COVID-19 mRNA vaccines and the large number of HCC patients treated with ICIs at tertiary centers, retrospective studies are now feasible. For instance, the NCT07341321 observational study is assessing whether vaccination within 3 months before ICI improves response (mRECIST), survival outcomes, and safety, and how cirrhosis severity, tumor characteristics or prior infection modify these effects (Grippin et al., 2025).

In CCA, the clinical application of LNP-based mRNA therapeutics remain at an early stage and requires further preclinical investigation to address tumor-specific limitations and to identify optimal antigenic targets for vaccine development. Early clinical studies in CCA have explored different immunotherapeutic strategies aimed at activating the immune system through distinct mechanisms. In particular, dendritic cell-based vaccines and peptide vaccines, administered either alone or in combination, have demonstrated the feasibility of translating immunotherapy into the clinical setting (Koido et al., 2014; Shimizu et al., 2012; Löffler et al., 2016).

Despite encouraging preliminary findings and advances *in silico* identification of potential tumor-associated antigens, the clinical implementation of mRNA vaccines in CCA remains challenging (Huang et al., 2021). Tumor-intrinsic immune escape mechanisms, together with significant interpatient heterogeneity, may limit therapeutic efficacy. Therefore, a deeper understanding of CCA immunobiology, including tumor heterogeneity and tumor–microenvironment interactions, is essential to ensure safe and effective clinical translation of mRNA-based immunotherapies.

### 7.2 Clinical applications of EV-RNA based strategy in HCC and biliary tumors

EVs exhibit a natural tropism for the liver, making them particularly suitable for therapeutic applications in HCC and biliary tumors. Tumor-derived EVs can deliver chemotherapeutic agents, miRNAs, or mRNA to malignant cells, thereby enhancing apoptosis, immune cell infiltration, and drug efficacy while reducing off-target toxicity (Bi et al., 2024; Słomka et al., 2020). EV-based vaccines or RNA therapeutics have demonstrated the ability to induce T cell responses, cytokine activation, and tumor suppression in preclinical hepatobiliary models (Li et al., 2025; Lu et al., 2024). As previously mentioned, EVs can also shape the TME by delivering proteins, RNAs, and metabolites that influence angiogenesis, ECM remodeling, EMT, chemoresistance, and immune evasion. Tumor EVs can deliver immune checkpoint proteins such as PD-L1 or immunosuppressive RNAs, thus impairing T cell activation and promoting regulatory T cell expansion (Liu et al., 2025; Gao et al., 2025). EV-mediated metabolic reprogramming in the liver can suppress drug metabolism, promoting tumor progression and limiting therapeutic efficacy (Wang G. et al., 2023; Wang Z. et al., 2023).

Patient-derived EVs loaded with chemotherapeutics or RNA cargo have demonstrated enhanced tumor targeting, immune activation, and anti-tumor efficacy in preclinical HCC models (Zhang J. et al., 2023) (Table 5). From a therapeutic perspective, inhibiting the tumorigenic pathways or disrupting EV biogenesis has been shown to sensitize tumors to chemotherapy and attenuate malignancy. In parallel, engineered EVs are being developed as targeted delivery vehicles for drugs and nucleic acids with the aim of improving treatment precision and overcome drug resistance mechanisms. These findings highlight the dual role of EVs as both mediators of disease progression and promising therapeutic tools, also in CCA management (Huang Z. et al., 2025; Lee et al., 2024; Rädler et al., 2023; Walker et al., 2019).

**TABLE 5:** Key signaling pathways HCC and CCA affected by EV therapeutic cargo delivery.

Pathway	Role in HCC	EV-delivered cargo	Therapeutic effect	References
Wnt/β-catenin	Tumor initiation, immune evasion, ICI resistance	siRNA-β-catenin, miR-34a	Reduced proliferation, restored immune sensitivity	Matsuda et al. (2019), He et al. (2023b)
Hippo–YAP/TAZ	Growth, stemness, metastasis	siRNA-YAP, miR-375	Suppressed tumor growth, EMT inhibition	He et al. (2023b), Wang et al. (2023c)
PI3K–AKT–mTOR	Survival, therapy resistance	miR-199a-3p, AKT siRNA	Increased apoptosis, TKI sensitization	Lou et al. (2020)
TGF-β/SMAD	EMT, fibrosis, immune suppression	miR-122, TGF-β siRNA	Reduced invasion, improved drug response	Nimitrungtawee et al. (2021)
VEGF/angiogenesis	Hypervascularization	Anti-VEGF siRNA, TKIs	Impaired tumor angiogenesis	Wang et al. (2024c)
PD-1/PD-L1 axis	Immune escape	miR-200, anti-PD-L1 siRNA	Enhanced ICI efficacy	Cheng et al. (2024)

Pathway	Role in CCA	EV-delivered Cargo	Therapeutic Effect	Reference(s)
TGF-β/SMAD	Promotes tumor progression, migration, and chemoresistance *via* SMAD activation	Latent TGF-β1, MMPs	MMP inhibitors block SMAD signaling; MEK inhibitors reduce EV secretion and sensitize cells to chemotherapy	Rodrigues-Junior et al. (2025)
MEK-ERK1/2	Enhances EV secretion and drug resistance	TGF-β-induced cargo	MEK pathway inhibition decreases EV release, improving chemotherapy response	Rodrigues-Junior et al. (2025)

However, direct evidence supporting EV–based therapeutic approaches in CCA remains limited, despite growing evidence of associations between EVs and this malignancy have been reported. For instance, EV-mediated crosstalk with cancer-associated fibroblasts (CAFs) has been implicated in the activation of TGF-β/SMAD signaling and fibrotic remodeling; chronic inflammation largely driven by IL-6/JAK/STAT3 signaling promotes tumor cell survival and immune evasion; EVs have been shown to transmit inflammatory cues within the TME; aberrant Wnt/β-catenin signaling further contributes to EMT and invasive behavior in CCA (Zhang N. et al., 2023; Wang J. et al., 2024). These findings highlight a substantial opportunity for the development of EV-based therapeutic platforms in CCA, which to date remain largely unexplored. As EVs are increasingly recognized as key regulators of oncogenic, stromal, and immune signaling, systematic preclinical investigation and translational validation of EV-enabled targeting strategies may provide new avenues for therapeutic intervention in this otherwise treatment-refractory malignancy.

## 8 Future strategies in hepatobiliary tumor-targeting strategies

Hybrid systems combining nanoparticles and EVs are increasingly being explored as promising platforms for hepatobiliary cancer therapy, as they integrate complementary advantages from both. Liposomes offer high drug loading capacity, biocompatibility, and ease of modification, while EVs provide intrinsic tumor-targeting capabilities and immune compatibility, especially important for liver cancers like HCC (Lehrich and Delgado, 2024; Samaei et al., 2024; Fulton and Najahi-Missaoui, 2023). For example, tumor-derived EV membranes fused with synthetic lipids create hybrid nanovesicles that facilitate siRNA delivery specifically to HCC cells by leveraging EVs’ tumor-homing properties and nanoparticles’ structural versatility, ultimately improving intracellular transport and gene silencing efficacy in preclinical models (Zhou et al., 2022).

These hybrids can circumvent endosomal degradation pathways and utilize intracellular transport routes such as the Golgi and endoplasmic reticulum, enhancing therapeutic payload delivery and antitumor effects *in vivo*. Additionally, stimuli-responsive liposomes functionalized with targeting ligands have been developed to enhance drug accumulation and release within the TME, which could be further potentiated through the incorporation of EV components for more precise delivery (Samaei et al., 2024; Mahmoud et al., 2022). Overall, hybrid liposome-EV systems represent a promising strategy to address the limitations associated with each platform individually, offering improved mRNA or siRNA delivery, enhanced tumor accumulation, and therapeutic efficacy in hepatobiliary cancers, though clinical translation remains at an early stage (Zhou et al., 2022; Lehrich and Delgado, 2024; Chen J. et al., 2024).

## 9 Conclusion

Despite major advances, significant challenges remain in applying RNA therapeutics and nanocarrier systems to hepatobiliary cancers, which are driven by late diagnosis, underlying liver dysfunction, complex multidisciplinary care, and the immunosuppressive TME. Additional hurdles include optimizing antigen selection, achieving precise immune modulation, improving drug loading and release kinetics, ensuring targeting specificity, and addressing scalable, reproducible manufacturing with rigorous safety evaluation and regulatory approval. Combining RNA-based therapies, including mRNA vaccines, with immune checkpoint inhibitors (ICIs) and other immunotherapies, together with continued refinement of nanocarrier design, represents a promising strategy to enhance clinical benefit. Within this landscape, LNPs and EVs offer complementary strengths for RNA delivery. LNPs provide a highly controllable and scalable platform, particularly suited for delivery of synthetic RNA cargos such as mRNA, supporting vaccine and protein replacement strategies. In contrast, EVs offer a biologically derived system with intrinsic biocompatibility, tissue tropism, and a natural capacity to transfer regulatory RNAs, enabling more nuanced modulation of the TME and intercellular signaling, although challenges in production, cargo loading, and standardization remain (Brezgin et al., 2024). Emerging hybrid systems, combining synthetic nanomaterials with EV-derived components, aim to integrate the engineering flexibility of LNPs with the biological functionality of EVs, potentially improving targeting, intracellular trafficking, and delivery precision. Rather than competing technologies, these platforms are increasingly viewed as complementary tools within a broader cell-free therapeutic framework, where platform selection, or combination, is guided by the biological context and therapeutic objective.
